# Quantifying the Impact of Ocrelizumab on Paramagnetic Rim Lesions in Multiple Sclerosis

**DOI:** 10.1002/acn3.70357

**Published:** 2026-03-10

**Authors:** Kimberly H. Markowitz, Neha V. Safi, Iliana Pliska‐Bloch, Ulrike W. Kaunzner, Ha Luu, Mert Sisman, Yi Wang, Thanh D. Nguyen, Sandra Hurtado Rúa, Susan A. Gauthier

**Affiliations:** ^1^ Department of Neurology Weill Cornell Medicine New York New York USA; ^2^ Department of Radiology Weill Cornell Medicine New York New York USA; ^3^ Mathematics and Statistics Cleveland State University Cleveland Ohio USA; ^4^ Feil Family Brain and Mind Institute Weill Cornell Medicine New York New York USA

**Keywords:** multiple sclerosis, ocrelizumab, quantitative susceptibility mapping

## Abstract

Paramagnetic rim lesions (PRLs) are a subset of chronic active multiple sclerosis (MS) lesions marked by iron‐laden microglia and macrophages. Ocrelizumab, a monoclonal antibody targeting CD20+ B cells, suppresses acute MS activity, but its effect on PRLs remains unclear. In a longitudinal study of 29 ocrelizumab‐treated patients with at least one PRL on quantitative susceptibility mapping (QSM), 97 PRLs were identified. Before treatment, PRLs showed higher QSM values than non‐PRLs (*p* = 0.001), indicating iron enrichment. After treatment, PRLs demonstrated a greater QSM reduction (*p* < 0.001), with an accelerated decline in susceptibility. These findings suggest ocrelizumab may attenuate iron‐related inflammation in PRLs.

## 1 Introduction

Paramagnetic rim lesions (PRLs), a subset of chronic active multiple sclerosis (MS) lesions, are associated with a more aggressive disease course [1, 2, 3], highlighting the need for effective therapies targeting these lesions [4]. Therapeutic approaches targeting B cells, such as ocrelizumab, have demonstrated substantial efficacy in modifying acute disease activity [5]. However, its potential secondary effect on chronic inflammation within PRLs remains unclear.

Quantitative susceptibility mapping (QSM), a technique that extracts tissue magnetic susceptibility by deconvolving the phase data from the gradient echo imaging (GRE), has been used to identify and quantify the underlying pathology of PRLs [6, 7, 8].

In this study, we aimed to determine whether treatment using ocrelizumab has a potential downstream effect on the CNS by altering the trajectory of chronic inflammation in PRLs, as measured using QSM.

## 2 Materials and Methods

### 2.1 Patient Cohort

This was a retrospective single‐arm study with a pre‐treatment run‐in phase to evaluate the impact of ocrelizumab on PRLs using QSM. Participants were selected retrospectively from an ongoing clinical and imaging database at the Weill Cornell MS Center. Inclusion criteria for this study were: (1) meeting the 2010 revised McDonald criteria [9]; (2) started ocrelizumab and had at least 1 year of prior imaging; (3) had at least one PRL defined on pre‐treatment QSM scan. Demographic and clinical data including age, sex, disease subtype, disease duration from the date of first symptom, previous disease‐modifying treatment (DMT), and expanded disability status scale (EDSS) were collected.

This study was reviewed and approved by Weill Cornell Medicine Institutional Review Board, which is an ethics standards committee on human experimentation. Patients provided informed consent for the use of their data.

### 2.2 MRI Acquisition and Reconstruction

Imaging was performed on 3T GE Signa HDxt and Siemens Magnetom Skyra MRI scanners. The MRI protocol consisted of sagittal 3D T1‐weighted (T1w) sequence with and without gadolinium, 2D T2‐weighted (T2w) fast spin echo, 3D T2w fluid attenuated inversion recovery (FLAIR), and axial 3D multi‐echo GRE sequence for QSM. Imaging parameters for each scanner are provided in the supplemental material. QSM was reconstructed from complex GRE images using a fully automated Morphology Enabled Dipole Inversion algorithm zero‐referenced to the cerebrospinal fluid across the entire brain (MEDI+0) [10].

For each time point, T1w image was brain‐extracted, intensity normalized and aligned to the FreeSurfer T1w conformed space (256 × 256 × 256 1 mm isotropic voxels) using FreeSurfer v6. Subsequently, T2w, T2w FLAIR, QSM images acquired at each time point were co‐registered to T‐1 FreeSurfer (time point immediately preceding treatment initiation) conformed space using ANTs image registration toolbox [11]. Further co‐registration details are provided in supplemental material.

### 2.3 Lesion Identification and Susceptibility Analysis

**FIGURE 1:**
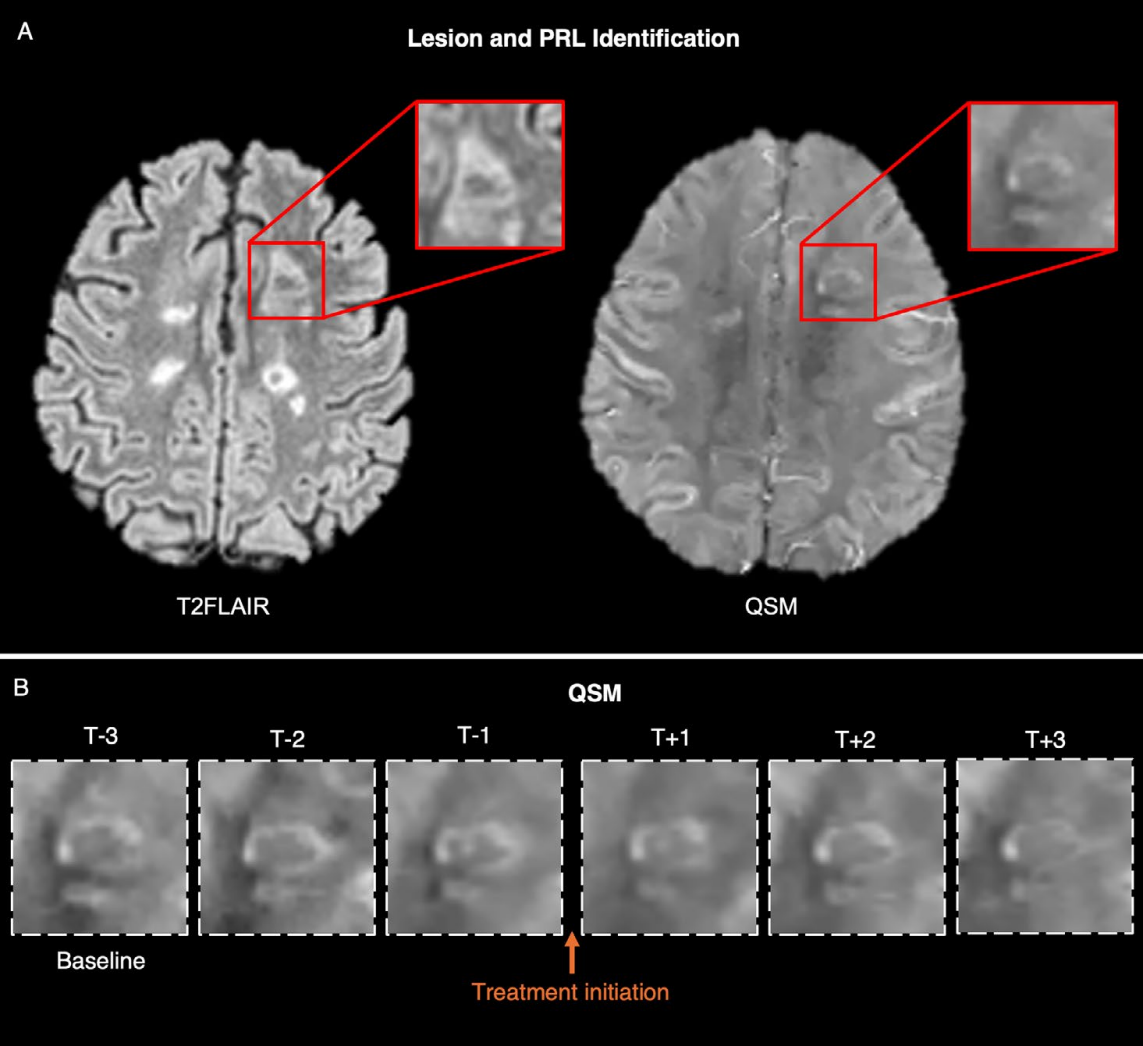
Longitudinal lesion tracking. Lesion masks were generated and edited based on the baseline T2FLAIR, which corresponds to the patient's earliest available scan. (A) Baseline T2FLAIR image and QSM image, with PRL lesion enlarged. (B) Temporal progression of the lesion was evaluated using QSM across all time points (pre‐ and post‐treatment) relative to treatment initiation.

MS lesions were automatically identified and segmented on the T2w FLAIR image [12] which was followed by manual editing. T1w and T2w images were referenced, as needed, during the editing process to define lesion boundaries, especially in the case of confluent lesions. Lesions below 15 mm [3] were removed. T2w FLAIR lesion labels were co‐registered to QSM, wherein the lesion boundary was further defined based upon the edge of the hyperintense rim on QSM at each time point. PRLs were identified based on the published consensus statement [13], using a consensus review, rim continuity of at least two‐thirds, and detection on two consecutive slices or two orthogonal planes. Acute enhancing lesions on T1w + Gd images were excluded from analysis. The mean QSM values were collected for each lesion on the baseline and across all co‐registered follow‐up QSM images (Figure 1).

## 3 Statistical Analysis

A mixed‐effects model estimated the effect of pre‐ and post‐treatment periods on QSM while accounting for multiple lesions per patient. A linear joint‐point regression model estimated the change points and slopes of the QSM trajectories for PRLs over time. A joint‐point model allows detecting structural changes in a continuous response variable by estimating breakpoints where the trajectory shifts [14]. We implemented both classical and Bayesian joint‐point regression models. Classical joint‐point estimation was performed using an iterative reweighted least squares (IRLS) algorithm, implemented via the segmented function [15]. Because this approach lacks direct uncertainty quantification for change points, we also applied a Bayesian model, which provides posterior distributions and credible intervals for all parameters, including the change points.

A detailed description of both the mixed‐effects and joint‐point regression models is presented in the supplemental material. The Bayesian output tables with estimates and 95% Highest Posterior Density Interval (HPDI) or credible intervals are also provided.

## 4 Results

### 4.1 Patient Cohort

This analysis included 29 patients, with 22 females (75.9%). Patient demographics and imaging details are present in Table 1. Majority of the cohort had relapsing–remitting MS (82.8%). The median number of PRLs per patient was 3.0 [2.0, 4.0]. Overall, 1458 lesions were included in the analysis, of which 97 (6.67%) were PRLs. EDSS remained stable across pre‐ and post‐treatment time points. The median number of pre‐treatment scans (5.0 [4.0, 6.0]) was similar to post‐treatment scans (4.0 [3.0, 6.0]).

**TABLE 1:** Demographics and imaging characteristics of the study cohort.

Number of patients	29
Sex, *n* (%)	
Males	7 (24.1)
Females	22 (75.9)
Disease category, *n* (%)	
CIS	2 (6.9)
RRMS	24 (82.8)
SPMS	2 (6.9)
PPMS	1 (3.4)
Disease duration (years), mean (SD)	6.79 (6.31)
Age, mean (SD)	40.5 (10.3)
EDSS at baseline, median [IQR]	1.25 [0.0, 2.0]
EDSS at treatment initiation, median [IQR]	1.50 [0.0, 3.0]
EDSS at last follow‐up, median [IQR]	1.25 [0.0, 3.6]
Prior DMT, *n* (%)	
Low efficacy	9 (31.0)
High efficacy	9 (31.0)
None	11 (37.9)
T2FLAIR lesion count per patient, median [IQR]	43.0 [26.0, 65.0]
PRL count per patient, median [IQR]	3.00 [2.00, 4.00]
Pre‐treatment follow‐up time (years), median [IQR]	4.20 [1.18, 5.62]
Post‐treatment follow‐up time (years), median [IQR]	4.09 [3.44, 5.81]
Number of pre‐treatment scans, median [IQR]	5.00 [4.00, 6.00]
Number of post‐treatment scans, median [IQR]	4.00 [3.00, 6.00]
First post‐treatment MRI (months), median [IQR]	9.84 [2.40, 12.12]

*Note:* Summary of baseline demographic, clinical, and imaging data for 29 patients included in the analysis. Values are presented as mean (SD) for normally distributed data and median [IQR] for non‐normally distributed data. Lower efficacy DMTs included: interferon beta‐1a, glatiramer acetate, and dimethyl fumarate. Higher efficacy DMTs included: fingolimod and natalizumab. Abbreviations: CIS, clinically isolated syndrome; DMT, disease‐modifying therapy; EDSS, Expanded Disability Status Scale; MRI, magnetic resonance imaging; PPMS, primary progressive multiple sclerosis; PRL, paramagnetic rim lesion; RRMS, relapsing–remitting multiple sclerosis; SPMS, secondary progressive multiple sclerosis.

### 4.2 Susceptibility Treatment Analysis for All Lesions

**FIGURE 2:**
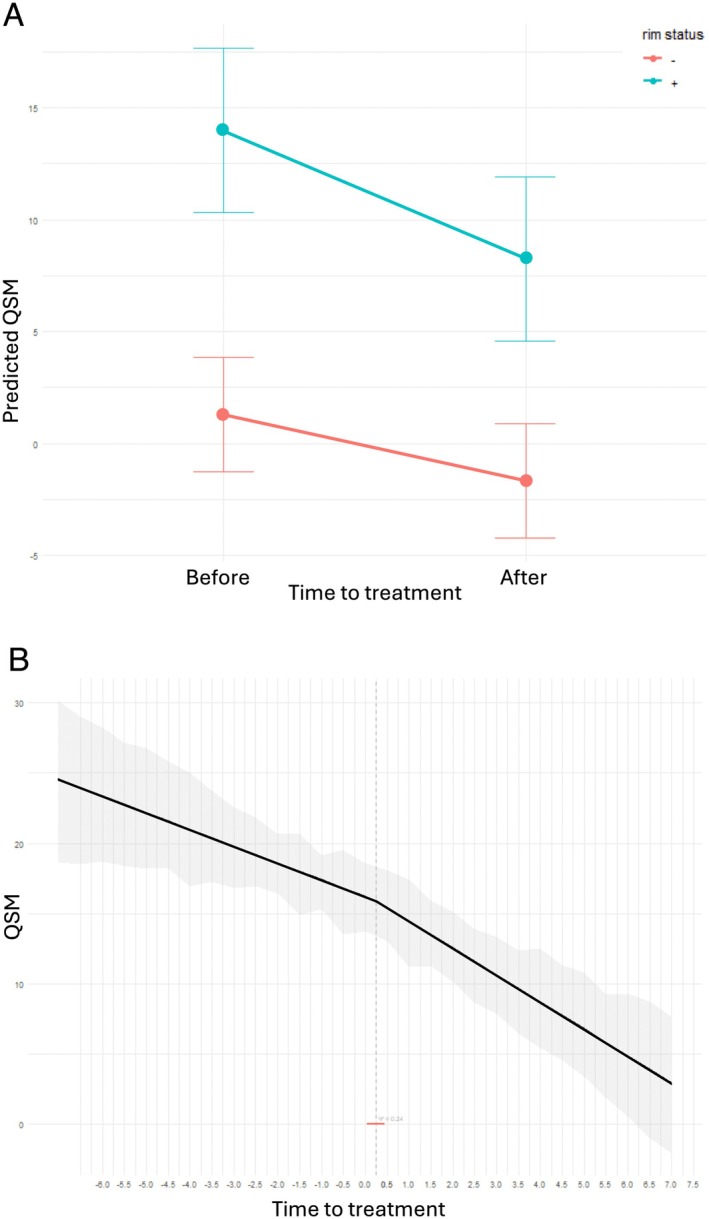
Mixed‐effects and bayesian models. (A) Mixed‐effects model of QSM values in PRLs (blue) and non‐PRLs (red) pre‐ and post‐treatment. Pre‐treatment QSMp was higher in PRLs (*p* = 0.001). Post‐treatment, PRLs showed an additional 2.9 ppb reduction beyond that in non‐PRLs (*p* < 0.001). (B) Bayesian join‐model estimated change point timing for PRLs. The shaded gray area shows the 95% confidence interval (not accounting for data correlation). A change point was estimated at 0.24 years (95% credible interval: Post‐treatment time 0 to month 5).

Mixed‐effects model estimated the effect of pre‐ and post‐treatment periods on QSM for PRLs and non‐PRLs. The mean pre‐treatment QSM for PRLs was 14.2 (95% CI: 10.5, 17.9) and was on average 12.96 ppb higher than non‐PRLs (*p* < 0.001). The post‐treatment reduction of QSM in PRLs was 2.9 ppb beyond the reduction observed in non‐PRLs (*p* < 0.001), (Figure 2A). The conditional *R* ^2^ for the model was 0.754.

### 4.3 Joint‐Point Analysis for PRLs

The Bayesian joint‐point regression model suggests that a notable change in the QSM trajectory for PRLs occurred approximately 3 months after treatment initiation (0.24 years) (Figure 2B). The 95% HPDI indicates that this change likely took place between treatment initiation and month 5. Prior to the change point, QSM for PRLs was decreasing at an average rate of −1.19 units per year (95% HPDI: −1.78, −0.59). Following the change, the decline accelerated to −1.92 units per year (95% HPDI: −2.47, −1.30). The model estimates the residual standard deviation (amount of unexplained variability) lies between 14.57 and 15.9 units with 95% probability. This indicates that while the model fits the data well, individual data points tend to deviate from model predictions by roughly 15 units.

## 5 Discussion

In this study, QSM was used to quantify the therapeutic impact of ocrelizumab on PRLs, a key contributor to CNS inflammation in MS [7].

Although ocrelizumab does not cross the blood brain barrier, peripheral B cell depletion may exert indirect effects on CNS inflammation through modulation of microglial and macrophage activity. Several studies have shown that B cell depletion may reduce antigen presentation, pro‐inflammatory cytokine release (TNF‐α, IL‐6, GM‐CSF), and CNS immune cell activation [16]. Since microglia are the dominant CNS‐resident antigen‐presenting cells in MS, their activation state is tightly linked to interactions with B cells, thus reduced B cell concentrations may consequently lead to decreased activation of these cells [17].

In contrast to our findings, prior QSM‐based studies assessing the effect of ocrelizumab on PRLs did not demonstrate a significant treatment‐response [18]. There is a significationt distinction between the study designs that may account for this discrepancy. Our study employed a *run‐in* design, in which each lesion served as its own control, whereas previous investigations used a non‐randomized comparator design without pre‐treatment imaging data. The absence of baseline information in those studies may have limited their ability to capture treatment‐related trajectory changes. By leveraging longitudinal imaging data from both pre‐ and post‐treatment periods, our approach enabled within‐subject comparisons that minimize between‐subject variability. Our design inherently controls for stable individual‐level confounders, thereby improving statistical efficiency and reducing random noise.

As in previous work [4], lesion age was unavailable for PRLs and represents a limitation; however, our joint‐point model offers a data‐driven approach to detecting shifts in lesion evolution over time. While PRLs are generally believed to transition to chronic inactive lesions over approximately seven years [19, 20], data from our group (unpublished) suggest that some persist for substantially longer. If our observed changes were solely attributable to age‐related decline, it would be unlikely to observe a consistent post‐treatment shift across lesions of varying ages. Importantly, lesion age can rarely be determined in clinical practice; overreliance on this parameter would limit the translational potential of PRLs as treatment‐response markers. Focusing instead on dynamic patterns, such as those captured by the joint‐point model, may provide greater insight into therapeutic effects. However, the interpretation of the joint‐point model requires caution. The estimated “shift” point should not be read as the exact time when a biological change occurred. Rather, it reflects the influence of later time points that drive the slope change. The apparent timing of this shift is also affected by variability in MRI scheduling (median: 9.8 months post‐treatment). Nevertheless, because the shift occurs after treatment initiation, it is supportive of a treatment‐related effect.

Although our study provides novel insights into the effects of ocrelizumab on chronic CNS inflammation in MS by leveraging QSM, several limitations must be acknowledged. The small cohort size limited our ability to control for patient demographics or clinical variables, such as disease category or previous disease‐modifying treatments, as well as our ability to assess treatment effects on disability. Moreover, disability scores remained stable over time, likely reflecting the small sample size and limiting our ability to link this finding to a clinically meaningful outcome. Larger studies are needed to determine whether dampening PRL inflammatory activity leads to measurable clinical improvement.

In conclusion, this study supports that iron content within PRLs declined after ocrelizumab initiation, suggesting reduced pro‐inflammatory activity. Future work should apply QSM in larger cohorts to better define ocrelizumab's effect on chronic inflammation in PRLs and explore how B cell depletion influences chronic CNS inflammatory activity. These findings highlight PRLs as potential imaging markers for monitoring treatment‐response in MS and support further longitudinal validation.

## Author Contributions

Study design: K.H.M., N.V.S., and S.A.G. Data acquisition: All authors. Data analysis and interpretation: K.H.M., T.D.N., S.H.R., and S.A.G. Manuscript preparation: K.H.M. and S.A.G. Critical revision of the manuscript for intellectual content: All authors. Study supervision and funding: S.A.G.

## Funding

This work was supported by Genentech.

## Conflicts of Interest

All authors have submitted their disclosure forms as part of submission. Dr. Kaunzner receives grant support from Genentech. Dr. Nguyen receives grant support from the National Multiple Sclerosis Society (NMSS) and NIH/NINDS. Dr. Hurtado Rúa receives grant funding from the NMSS and NIH/NIDS. Dr. Gauthier receives grant support from the NMSS, NIH/NINDS, Genentech and receives consulting fees from Novartis and Biogen. Sponsors did not have a role in the study design, in the collection, analysis, interpretation of data, in the writing of the report, or in the decision to submit the article for publication.

## Supporting information


**Figure S1:** QSM convergence diagnosis. Left: Marginal posterior distributions and trace plots for a subset of model parameters (organized by row), with three MCMC chains distinguished by color. Diagnostics indicate satisfactory mixing and convergence across chains, suggesting stable posterior sampling. Right: Joint posterior density plot illustrating the bivariate relationship between the estimated change point and the slope parameter of the subsequent segment, highlighting potential posterior dependency or trade‐offs between these estimates. cp_1 represents the change points, time.to.trt1 represents the first slope and time.totr2 indicates the slope after change point.

**Table S1:** Fixed effects estimate from the QSM linear mixed‐effects model.
**Table S2:** Bayesian posterior estimates for model parameters corresponding to QSM.
**Table S3:** Bayesian hypothesis testing.

## Data Availability

The data that support the findings of this study are available from the corresponding author upon reasonable request.
