# Supplementary figures and images for: Quantifying the Impact of Ocrelizumab on Paramagnetic Rim Lesions in Multiple Sclerosis
Source: Ann Clin Transl Neurol. 2026 Mar 10;13(7):1482–7. doi: 10.1002/acn3.70357 (PMC13358547; doi:10.1002/acn3.70357)

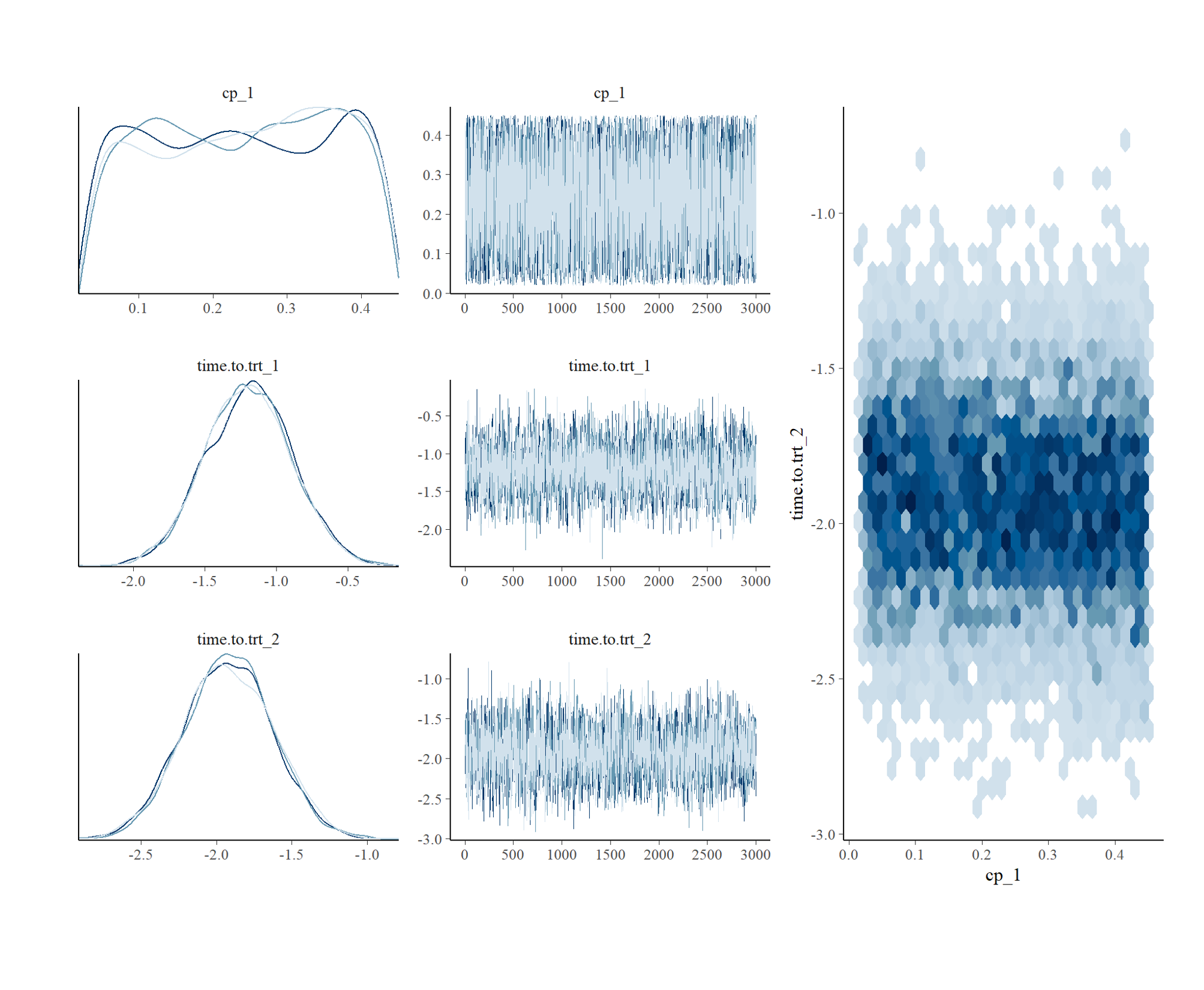

Supplement: Supplementary file 1 — Figure S1: QSM convergence diagnosis. Left: Marginal posterior distributions and trace plots for a subset of model parameters (organized by row), with three MCMC chains distinguished by color. Diagnostics indicate satisfactory mixing and convergence across chains, suggesting stable posterior sampling. Right: Joint posterior density plot illustrating the bivariate relationship between the estimated change point and the slope parameter of the subsequent segment, highlighting potential posterior dependency or trade‐offs between these estimates. cp_1 represents the change points, time.to.trt1 represents the first slope and time.totr2 indicates the slope after change point. [file ACN3-13-1482-s001.tiff]
