# Supplementary material for: Quantifying the Impact of Ocrelizumab on Paramagnetic Rim Lesions in Multiple Sclerosis
Source: Ann Clin Transl Neurol. 2026 Mar 10;13(7):1482–7. doi: 10.1002/acn3.70357 (PMC13358547; doi:10.1002/acn3.70357)
Supplement: Supplementary file 2 — Table S1: Fixed effects estimate from the QSM linear mixed‐effects model. Table S2: Bayesian posterior estimates for model parameters corresponding to QSM. Table S3: Bayesian hypothesis testing. [file ACN3-13-1482-s002.docx]

**Supplemental Material**

**Title: Quantifying the Impact of Ocrelizumab on Paramagnetic Rim Lesions in Multiple Sclerosis**

1. **Detailed MRI Protocol**

*The Siemens scanning protocol consisted of the following sequences*: 1) 3D sagittal T1-weighted (T1w) MPRAGE: Repetition Time (TR)/Echo Time (TE)/Inversion Time (TI) = 2300/2.3/900 ms, flip angle (FA) = 8°, GRAPPA parallel imaging factor (R) = 2, voxel size = 1.0 × 1.0 × 1.0 mm^3^; 2) 2D axial T2-weighted (T2w) turbo spin echo: TR/TE = 5840/93 ms, FA = 90°, turbo factor = 18, R = 2, number of signal averages (NSA) = 2, voxel size = 0.5 × 0.5 × 3 mm^3^; 3) 3D sagittal fat-saturated T2w fluid attenuated inversion recovery (FLAIR) SPACE: TR/TE/TI = 8500/391/2500 ms, FA = 90°, turbo factor = 278, R = 4, voxel size = 1.0 × 1.0 × 1.0 mm^3^.

*The GE scanning protocol consisted of the following sequences:* 1) 3D sagittal T1w BRAVO: TR/TE/TI =  8.8/3.4/450 ms, FA = 15°, voxel size = 1.2 × 1.2 × 1.2 mm^3^, ASSET parallel imaging acceleration factor (R) = 1.5; 2) 2D axial T2w fast spin echo: TR/TE = 5267/86 ms, FA = 90°, echo train length = 100, number of excitations (NEX) = 2, voxel size = 0.6 x 0.9 x 3.0 mm^3^ ; 3) 3D sagittal T2w FLAIR CUBE: TR/TE/TI = 5000/139/1577 ms, FA = 90°, ETL = 162, R = 1.6, voxel size = 1.2 × 1.2 × 1.2 mm^3^.

Both scanners had similar parameters for the axial 3D multi-echo GRE sequence for QSM: axial field of view (FOV) = 24 cm, TR/TE1/ΔTE = 48.0/6.3/4.1 ms, number of TEs = 10, FA = 15°, R = 2, voxel size = 0.75 x 0.93 x 3 mm^3^, scan time = 4.2 min. The harmonized QSM imaging protocol has demonstrated high reproducibility across different scanner vendors^1-3^. 16/29 (55%) were scanned on both GE and Siemens; 13/29 (45%) patients were scanned on Siemens only.

1. **Image co-registration**

For each time point, T1w image was brain-extracted, intensity normalized and aligned to the FreeSurfer T1w conformed space (256 x 256 x 256 1 mm isotropic voxels) using FreeSurfer v6.^11^. At each time point, images were registered cross-sectionally to the FreeSurfer T1 image using a rigid-body transformation model (translation and rotation) and mutual information similarity metric. For the QSM image, the corresponding echo-combined GRE magnitude image, which is already aligned with the QSM, was used to determine the transformation. For longitudinal registration, the FreeSurfer T1 image at each time point was aligned to the baseline (image immediately preceding treatment) FreeSurfer T1 image using rigid-body transformation with mutual information similarity metric, followed by non-linear deformation using ANTs^4^ with cross-correlation similarity metric. The resulting transformation matrix and warp field were concatenated with the cross-sectional transformation to bring each image to the baseline FreeSurfer T1 space. Spline interpolation was used in all registrations. This automated registration approach was applied consistently to all images, and the registration outputs were visually inspected in ITK-SNAP for quality control

1. ***Statistical Analysis***

We used a model-based approach to study the trajectory of chronic inflammation in PRLs. The model-based approach includes a mixed effects model and a joint-point regression model.

**A. Mixed Effects Model**

We used mixed effects models^5^ to estimate the effect of pre- and post-treatment periods on QSM biomarkers while accounting for multiple lesions per patient. The model was fitted using restricted maximum likelihood (REML), and its fit was assessed through likelihood ratio tests, AIC, and residual diagnostics. Random intercept variance estimates were examined to ensure appropriate within-subject correlation modeling. We estimated the following questions:

${QSM}_{ij}=\beta_{1} {TRT}_{ij}+ \beta_{2} {PRL}_{ij}+ + \beta_{12} {TRT}_{ij}* {PRL}_{ij}+b_{oj}+b_{oij}+\epsilon_{ij}$

Where:

- ${QSM}_{ij}$is the QSM susceptibility (response) for lesion i patient j.
- $\beta_{1}$ ​ is the fixed effect of pre- and post-treatment effect (${TRT}_{ij}$) for lesion i, patient j.
- $\beta_{2}$​ is the fixed effect of lesion type (${PRL}_{ij}=1$ if lesion i, patient j is a PRL lesion versus 0 otherwise)
- $\beta_{12}$​ is the fixed effect of the interaction between pre- and post- treatment status (${TRT}_{ij}$) and lesion type (${PRL}_{ij}$)

- $b_{oj} \sim N(0, \sigma_{subj}^{2})$ is the random intercept for patient j
- $b_{oij} \sim N(0, \sigma_{lesion}^{2})$ is the random intercept for lesion i within patient j
- $\epsilon_{ij} \sim N(0, \sigma^{2}$) is the residual error

Mixed-effects models offer several key advantages, especially when analyzing data with complex structures. One major benefit is their ability to account for both fixed effects (pre- and pos- treatment and lesion type) and random effects (multiple lesions per patient). By modeling random variation appropriately, mixed-effects models improve the accuracy of estimates and reduce the risk of inflated Type I error rates. They also handle our unbalanced (different number of lesions per patient) data well without requiring listwise deletion. Overall, mixed-effects models provide a flexible and powerful framework for analyzing QSM data at the lesion level.

**B. QSM Mixed Effects Model Estimates**

Diagnostic checks of the linear mixed-effects models (Table S1) revealed that the residuals were approximately normally distributed, as evidenced by Q-Q plots, and showed no systematic patterns when plotted against fitted values, supporting the assumption of homoscedasticity. No influential outliers were detected based on standardized residuals. The model explanatory power was assessed using the conditional $R^{2}$ , which accounts for both fixed and random effects. The conditional $R^{2}$ for the model was 0.754, indicating that the model explains 75% of the variance in the outcome after accounting for both patient-level and lesion-level variation. Overall, all residual diagnostics and fit indices suggest that both models appropriately capture the structure of the data. A separate model including scanner type showed no meaningful effect on the results.

**C. Joint-point regression models**

A linear joint-point regression model was used to estimate the change points and slopes of the QSM trajectories for PRLs over time.^6^ This joint-point model enables the detection of structural changes in the time variable ($t_{ij}$), identifying breakpoints where the trajectory undergoes a shift. In this model, time is defined in years (or fractions of a year), with negative values representing the period before treatment and positive values indicating the period after treatment.

${QSM}_{ij}\left( t_{ij} \right)=\beta_{0}+b_{oj}+\beta_{1} t_{ij}+\beta_{2} {( t_{ij}- \tau)}_{+}+\epsilon_{ij}$

${QSM}_{ij}$is the QSM susceptibility (response) for lesion i patient j at time t. ${QSM}_{ij}$is the QSM susceptibility (response) for lesion i patient j at time t. $\beta_{0}$ is the fixed intercept, $b_{oj} \sim N(0, \sigma_{subj}^{2})$ is the random intercept for patient j, $\beta_{1}$is the slope before the change point, $\beta_{2}$ is the change in slope after the change point, τ represents the change point, ${( t_{ij}- \tau)}_{+}= max\{0, t_{ij}- \tau\}$ represents the “hinge” or segmented term, active only after the change point, and $\epsilon_{ij} \sim N(0, \sigma^{2}$) is the residual error.

We implemented both a classical and Bayesian joint-point regression model. The classical implementation estimated change-points using an iterative reweighted least squares algorithm (IRLS), as implemented in the segmented function.^7^ Initial linear regression models were fitted using the ordinary least squares (OLS) method, and change-point estimates were refined using the Davies test for significance.^8, 9^ Model convergence was assessed through visual inspection of residual plots and diagnostic criteria, including Akaike Information Criterion (AIC) for model selection. Model assumptions were validated by checking for normality of residuals (Shapiro-Wilk test), homoscedasticity (Breusch-Pagan test), and absence of autocorrelation (Durbin-Watson test).

A disadvantage of the classical approach is that it does not provide direct uncertainty quantification for the estimated change points. To address this limitation, we implemented a Bayesian change-point model using the mcp package in R.^10^ The Bayesian approach allows for posterior distribution estimation of all parameters, including the change points, enabling probabilistic interpretation and credible intervals for the breakpoints. The Bayesian model parameters are estimated via Markov Chain Monte Carlo (MCMC) sampling. Prior distributions were assigned to model parameters using a Conjugate-Empirical Bayes approach,^11^ including normal priors for regression coefficients and a uniform prior for change-point locations. Hyperparameters for the prior distributions were estimated from the classical IRLS estimates. Model inference was conducted using Hamiltonian Monte Carlo (HMC). Convergence was evaluated through multiple diagnostics, including the Gelman-Rubin diagnostic, effective sample size (ESS), the Rhat statistic, and visual inspection of trace plots. Three independent HMC chains were executed in parallel to assess sampling stability and consistency across chains.

All analysis was performed using R: A language and environment for statistical computing using the lm(), segmented(), and mcp() functions. ^12^

**D. Bayesan Joint-point regression model Estimates**

Posterior estimates represent the central tendency of the model parameters after accounting for prior distributions, while the HPDI provides the range within which the true parameter value lies with 95% probability. The Rhat statistic is used to assess convergence in Bayesian models by comparing the variance within chains to the variance between chains. An Rhat value close to 1 indicates that the model has converged, suggesting that the chains are mixing well and exploring the parameter space effectively (Table S2). Prior information reflects the EB estimates, which were derived from classical IRLS fitting before the Bayesian updating process.

**E. Bayesian Hypothesis Testing**

Hypotheses about parameter values can be tested using the p and Bayes Factors (BF) calculated from the Savage-Dickey density ratio.^13^ A value of 𝑝 close to 1 indicates strong evidence in favor of the specified hypothesis. A Bayes factor (BF) greater than 1 strengthens belief in the point hypothesis by a factor of approximately ‘BF’ relative to prior beliefs (Table S3). Conversely, the inverse (i.e., 1 − p and 1/BF) is the evidence in favor of the alternative.

**E. Bayesian Convergence Diagnosis**

Model inference was conducted using Hamiltonian Monte Carlo (HMC). Convergence was evaluated through multiple diagnostics, including the Gelman-Rubin, effective sample size (ESS), the Rhat statistic, and visual inspection of trace plots. Three independent HMC chains were executed in parallel to assess sampling stability and consistency across chains (Figure S1)

**References**

1. Deh K, Nguyen TD, Eskreis-Winkler S, et al. Reproducibility of quantitative susceptibility mapping in the brain at two field strengths from two vendors. J Magn Reson Imaging. 2015 Dec;42(6):1592-600.

2. Naji N, Lauzon ML, Seres P, et al. Multisite reproducibility of quantitative susceptibility mapping and effective transverse relaxation rate in deep gray matter at 3 T using locally optimized sequences in 24 traveling heads. NMR Biomed. 2022 Nov;35(11):e4788.

3. Lancione M, Bosco P, Costagli M, et al. Multi-centre and multi-vendor reproducibility of a standardized protocol for quantitative susceptibility Mapping of the human brain at 3T. Phys Med. 2022 Nov;103:37-45.

4. Tustison NJ, Cook PA, Klein A, et al. Large-scale evaluation of ANTs and FreeSurfer cortical thickness measurements. NeuroImage. 2014 Oct 1;99:166-79.

5. Bates D, Mächler M, Bolker B, Walker S. Fitting linear mixed-effects models using lme4. Journal of Statistical Software. 2015;67(1):1-48.

6. Muggeo VM. Estimating regression models with unknown break-points. Stat Med. 2003 Oct 15;22(19):3055-71.

7. Muggeo VMR. Segmented: An R package to fit regression models with broken-line relationships. The R Journal. 2008;8(1):20-5.

8. Davies RB. Hypothesis testing when a nuisance parameter is present only under the alternative. Biometrika. 1987;74(1):33-43.

9. Muggeo VM. Interval estimation for the breakpoint in segmented regression: A smoothed score-based approach. Australian & New Zealand Journal of Statistics. 2017;59(3):311-22.

10. Lindeløv JK. mcp: An R package for Bayesian regression with change points. Journal of Open Source Software. 2020;5(53).

11. Efron B. Large-Scale Inference: Empirical Bayes Methods for Estimation, Testing, and Prediction. . Institute of Mathematical Statistics Monographs Cambridge, UK: Cambridge University Press.; 2010.

12. Team RC. R: A language and environment for statistical computing. Vienna, Austria: R Foundation for Statistical Computing; 2023.

13. Bürkner PC. Brms: An R package for Bayesian multilevel models using Stan. Journal of Statistical Software2017. p. 1-28.

**Supplemental Figure Legend:**

**Figure S1. QSM Convergence Diagnosis.** Left: Marginal posterior distributions and trace plots for a subset of model parameters (organized by row), with three MCMC chains distinguished by color. Diagnostics indicate satisfactory mixing and convergence across chains, suggesting stable posterior sampling. Right: Joint posterior density plot illustrating the bivariate relationship between the estimated change point and the slope parameter of the subsequent segment, highlighting potential posterior dependency or trade-offs between these estimates. cp_1 represents the change points, time.to.trt1 represents the first slope and time.totr2 indicates the slope after change point.

**Supplemental Tables:**

**Table S1.** **Fixed effects estimate from the QSM linear mixed-effects model.**

| **Parameter** | **Estimate** | **95% C.I** | **p-value** |
| --- | --- | --- | --- |
| pre_treatment effect | 1.29 | (-1.26, 3.84) | 0.321 |
| post_treatment effect | -1.65 | (-4.20 , 0.89) | 0.203 |
| PRLs | 12.69 | (9.80 , 15.59) | <0.001 |
| post_treatment effect x PRLs | -2.78 | (-4.00 , -1.56 ) | <0.001 |

Reported values include the estimated coefficients (Estimate), 95% confidence intervals (95% C.I), and associated p-values (p). Confidence intervals reflect the uncertainty in the parameter estimates. Statistical significance was assessed at the 0.05 level.

**Table S2*.*** **Bayesian posterior estimates for model parameters corresponding to QSM.**

| Parameter | Posterior Mean | HPDI Lower | HPDI Upper | Rhat | EB Prior  Mean | EB Prior  SD |
| --- | --- | --- | --- | --- | --- | --- |
| τ | 0.2396 | 0.0434 | 0.4492 | 1.000 | 0.20 | 0.12 |
| $\beta_{0}$ | 16.2124 | 14.7328 | 17.6454 | 1.003 | 23.75 | 16 |
| β₁ | -1.1948 | -1.7760 | -0.5875 | 1.002 | -1.00 | 1.5 |
| β₂ | -1.9194 | -2.4703 | -1.3021 | 1.002 | -1.80 | 1.5 |
| σ (Residual ) | 15.2634 | 14.5701 | 15.9085 | 1.000 | 25 | 10 |

The table reports the posterior mean estimates, the 95% Highest Posterior Density Interval (HPDI), the Rhat statistics as convergence diagnostic measurement, and prior information from the empirical Bayes (EB) estimates based on the classical model. The prior means were derived from estimates obtained through a classical model, while the prior standard deviations (SD) were inflated to ensure a flatter prior distribution.

**Table S3.** **Bayesian Hypothesis Testing.**

| Parameter | Posterior Mean | HPDI Lower | HPDI Upper | Rhat | EB Prior  Mean | EB Prior  SD |
| --- | --- | --- | --- | --- | --- | --- |
| τ | 0.2396 | 0.0434 | 0.4492 | 1.000 | 0.20 | 0.12 |
| $\beta_{0}$ | 16.2124 | 14.7328 | 17.6454 | 1.003 | 23.75 | 16 |
| β₁ | -1.1948 | -1.7760 | -0.5875 | 1.002 | -1.00 | 1.5 |
| β₂ | -1.9194 | -2.4703 | -1.3021 | 1.002 | -1.80 | 1.5 |
| σ (Residual ) | 15.2634 | 14.5701 | 15.9085 | 1.000 | 25 | 10 |

All 𝑝 are greater than 0.92 indicating strong evidence in favor of the specified hypothesis. The fact that all BF>1 strengthens belief in the point hypothesis by a factor of approximately ‘BF’ relative to prior beliefs
